# Gut Microbiota-Targeted Nutrition for Healthy Aging: Mechanistic Roles of Polyphenols and Dietary Fiber in Geroscience

**DOI:** 10.3390/nu18152478

**Published:** 2026-07-31

**Authors:** Agata Kryczyk-Poprawa, Elżbieta Rząsa-Duran, János Tamás Varga, Andrea Lehoczki, Virág Zábó, Vince Fazekas-Pongor, Dávid Major, Tamás Csípő, Ágnes Szappanos, Ágnes Lipécz, Mónika Fekete

**Affiliations:** 1 Department of Inorganic Chemistry and Pharmaceutical Analytics, Faculty of Pharmacy, Jagiellonian University Medical College, 31-008 Krakow, Poland; 2 Branch in Krakow-Hospital Pharmacy, Maria Sklodowska-Curie National Research Institute of Oncology, Garncarska 11 Str., 31-115 Krakow, Poland; duranela@poczta.onet.pl; 3 Department of Pulmonology, Semmelweis University, 1083 Budapest, Hungary; varga.janos.tamas@semmelweis.hu; 4 Institute of Preventive Medicine and Public Health, Faculty of Medicine, Semmelweis University, 1085 Budapest, Hungary; ceglediandi@freemail.hu (A.L.); zabo.virag@semmelweis.hu (V.Z.); pongor.vince@semmelweis.hu (V.F.-P.); major.david@semmelweis.hu (D.M.); csipo.tamas@semmelweis.hu (T.C.); lipecz.agnes@semmelweis.hu (Á.L.); 5 Health Sciences Division, Doctoral College, Semmelweis University, 1085 Budapest, Hungary; 6 Fodor Center for Prevention and Healthy Aging, Semmelweis University, 1085 Budapest, Hungary; 7 Heart and Vascular Center, Semmelweis University, 1122 Budapest, Hungary; drszappanos@gmail.com

**Keywords:** gut microbiota, polyphenols, dietary fiber, healthy aging, inflammaging, immunosenescence, gut–brain axis, gut–muscle axis, short-chain fatty acids, geroscience, longevity

## Abstract

Age-related alterations in the gut microbiota contribute to chronic low-grade inflammation, immune dysregulation, metabolic dysfunction, frailty, sarcopenia, and cognitive decline. Dietary polyphenols and fermentable fiber modulate microbial composition and metabolism, promoting the production of bioactive metabolites, including short-chain fatty acids, secondary bile acids, indole derivatives, and urolithins, which regulate intestinal barrier integrity, immune homeostasis, mitochondrial function, and gut–organ communication. This narrative review critically synthesizes evidence from experimental studies, observational cohorts, randomized controlled trials, systematic reviews, and meta-analyses to examine microbiota-mediated mechanisms linking these dietary components to healthy aging within the geroscience framework. Although mechanistic evidence is compelling, translation into clinically meaningful aging outcomes remains limited because most intervention studies are small and heterogeneous and primarily rely on surrogate biomarkers. Current evidence supports polyphenol- and fiber-rich dietary patterns as biologically plausible strategies for promoting healthy aging through modulation of the gut microbiota; however, establishing causal relationships will require standardized microbiome methodologies, validated microbiome-derived biomarkers, integrated multi-omics approaches, and adequately powered longitudinal studies and randomized controlled trials.

## 1. Introduction

Aging is a multifactorial biological process characterized by progressive functional decline accompanied by chronic low-grade inflammation (inflammaging), immune dysregulation, metabolic dysfunction, frailty, sarcopenia, and cognitive impairment [1]. According to the geroscience hypothesis, aging is driven by a network of interconnected biological hallmarks, including genomic instability, epigenetic alterations, loss of proteostasis, mitochondrial dysfunction, cellular senescence, stem cell exhaustion, deregulated nutrient-sensing pathways, chronic inflammation, impaired intercellular communication, and alterations in the gut microbiome. Rather than acting independently, these hallmarks interact through complex immunometabolic and stress-response networks, collectively contributing to functional decline and increased susceptibility to chronic diseases. Emerging evidence suggests that the gut microbiota and its metabolites influence multiple hallmarks of aging simultaneously, thereby providing a mechanistic link between nutrition, metabolism, immunity, and healthy aging.

Increasing evidence suggests that the gut microbiota contributes to several biological processes involved in aging and age-related diseases, although many mechanistic relationships remain incompletely understood [2]. Gut microbial composition and function are continuously shaped by diet, physical activity, medication use, environmental exposures, and other lifestyle factors throughout life [3].

Compared with younger adults, older individuals frequently exhibit altered gut microbial diversity and metabolic activity, including reduced abundance of short-chain fatty acid (SCFA)-producing microorganisms. These alterations have been associated with impaired intestinal homeostasis and changes in the production of microbiota-derived metabolites that participate in immune and metabolic regulation [4,5,6].

Age-associated gut microbial dysbiosis has emerged as a central contributor to inflammaging and immunosenescence. Reduced microbial diversity, depletion of beneficial SCFA-producing bacteria, and expansion of pathobionts promote increased intestinal permeability, allowing microbial products such as lipopolysaccharide (LPS), peptidoglycans, and other pathogen-associated molecular patterns (PAMPs) to translocate into the systemic circulation. These molecules activate innate immune signaling through Toll-like receptors (TLRs), particularly Toll-like receptor 4 (TLR4), nucleotide-binding oligomerization domain-like receptors (NLRs), and the NOD-, LRR- and pyrin domain-containing protein 3 (NLRP3) inflammasome, resulting in sustained production of pro-inflammatory cytokines including interleukin-1β, interleukin-6, and tumor necrosis factor-α. Persistent activation of these pathways reinforces chronic low-grade inflammation, accelerates immunosenescence, and contributes to the pathogenesis of metabolic disorders, cardiovascular disease, neurodegeneration, frailty, and other age-related conditions.

Concurrently, aging is associated with progressive deterioration of intestinal barrier integrity. Structural and functional alterations—including reduced expression of tight-junction proteins, including occludin, claudins, and zonula occludens-1 (ZO-1); impaired mucus production; diminished secretion of antimicrobial peptides by Paneth cells; and epithelial dysfunction—compromise the gut barrier and facilitate microbial translocation. Loss of barrier function amplifies systemic inflammation, disrupts immune homeostasis, and alters host–microbiome communication through the gut–brain, gut–muscle, gut–liver, and gut–immune axes. Preservation of intestinal barrier integrity has therefore emerged as a major therapeutic target for promoting healthy aging.

Among modifiable environmental factors, diet is one of the strongest determinants of gut microbial composition and function [5,7]. Dietary patterns rich in polyphenols and fermentable fiber have been associated with favorable changes in microbial metabolism, although responses vary considerably according to baseline microbiome composition, dietary habits, host characteristics, and study design [8]. These dietary components not only reshape microbial ecology but also increase the production of bioactive metabolites—including SCFAs, indole derivatives, secondary bile acids, urolithins, and phenolic metabolites—that regulate mitochondrial quality control, oxidative stress responses, epigenetic remodeling, nutrient-sensing pathways involving AMP-activated protein kinase (AMPK), the mechanistic target of rapamycin (mTOR), and sirtuins, as well as immune function. Through these mechanisms, microbiota-derived metabolites represent key molecular mediators linking diet to several biological hallmarks of aging. Advances in microbiome research, metabolomics, and precision nutrition have further stimulated interest in individualized dietary approaches that account for interindividual variability in host–microbiome interactions [9,10].

Despite rapid progress in microbiome research, important uncertainties remain. Although numerous studies have identified associations between gut microbial composition and aging-related phenotypes, relatively few have demonstrated causal relationships or clinically meaningful improvements in healthspan. Furthermore, differences in sequencing technologies, metabolomic platforms, dietary assessment methods, participant characteristics, and analytical pipelines substantially limit comparability across studies and contribute to inconsistent findings [11,12,13]. Additional challenges include substantial interindividual variability in microbiome composition, microbial metabolic capacity, and dietary responsiveness, underscoring the need for standardized microbiome methodologies, validated microbiome-derived biomarkers, and integrated multi-omics approaches to establish causal mechanisms and enable precision nutrition strategies for healthy aging.

**Figure 1:**
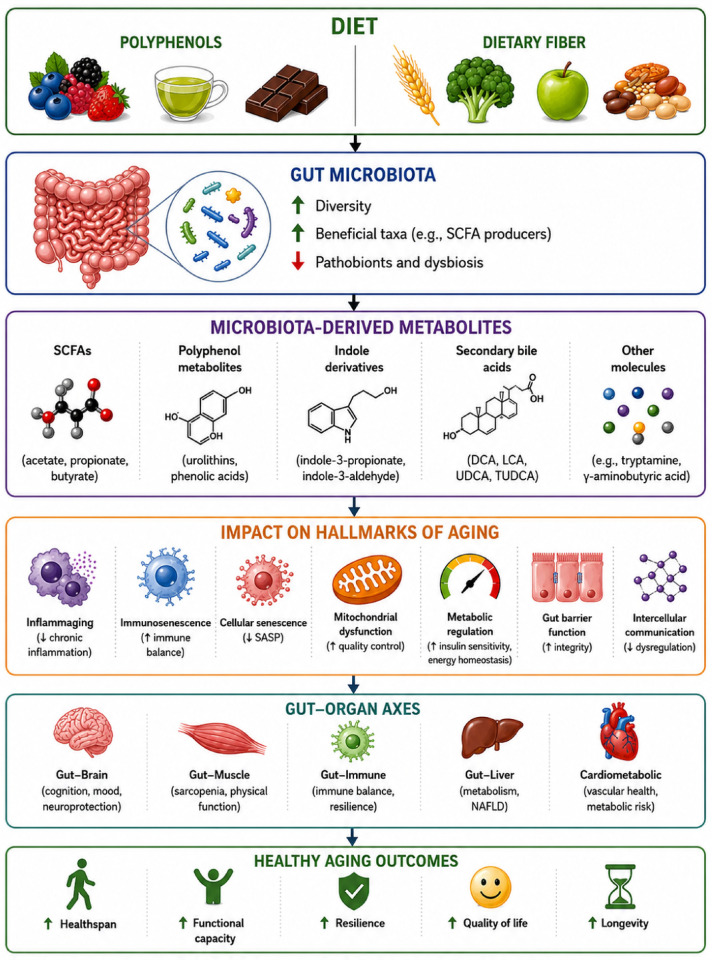
Schematic overview of the proposed interactions between dietary polyphenols, dietary fiber, the gut microbiota, microbiota-derived metabolites, hallmarks of aging, gut–organ axes, and healthy aging outcomes. Green upward arrows (↑) indicate an increase, enhancement, or improvement, whereas red downward arrows (↓) indicate a reduction or suppression. Black downward arrows represent the proposed directional progression between the successive levels of the conceptual framework. SCFAs, short-chain fatty acids; SASP, senescence-associated secretory phenotype; DCA, deoxycholic acid; LCA, lithocholic acid; UDCA, ursodeoxycholic acid; TUDCA, tauroursodeoxycholic acid; NAFLD, non-alcoholic fatty liver disease. This figure is conceptual and intended for illustrative purposes; the arrows represent proposed biological links and do not imply direct causal relationships.

This narrative review synthesizes current evidence on the interactions among dietary polyphenols, fermentable fiber, the gut microbiota, and biological aging from a geroscience perspective. It focuses on age-related changes in gut microbial ecology, microbiota-derived metabolites, and gut–organ communication while distinguishing mechanistic evidence derived from experimental models from findings supported by human intervention studies. Particular emphasis is placed on the molecular mechanisms through which microbiota-derived metabolites influence the biological hallmarks of aging, including mitochondrial dysfunction, inflammaging, immunosenescence, impaired nutrient sensing, and epithelial barrier dysfunction. To integrate these interconnected mechanisms, we propose the Polyphenol–Fiber–Microbiota–Metabolite–Hallmarks of Aging Axis (Figure 1), which provides a comprehensive mechanistic framework for understanding how microbiota-mediated nutritional interventions may modulate the biological hallmarks of aging and promote healthy aging.

## 2. Methods

This narrative review critically evaluates current evidence on microbiota-targeted nutritional strategies for healthy aging, with a particular focus on dietary polyphenols, fermentable fiber, and microbiota-derived metabolites. A structured literature search was conducted in PubMed/MEDLINE, Scopus, and Web of Science between January and March 2026. Approximately 18,900 records were identified across the three databases. Following title and abstract screening, full-text assessment, and removal of duplicates and non-relevant publications, approximately 290 studies were included in the final narrative synthesis.

The search strategy combined controlled vocabulary and free-text terms related to gut microbiota, nutrition, and aging, including gut microbiota, gut microbiome, polyphenols, dietary fiber, healthy aging, geroscience, inflammaging, immunosenescence, gut–brain axis, gut–muscle axis, short-chain fatty acids, microbiota-derived metabolites, longevity, and age-related diseases, using the Boolean operators AND and OR. Reference lists of eligible articles were also manually screened to identify additional relevant studies.

Priority was given to studies published between 2016 and 2026 to reflect recent advances in microbiome research, metabolomics, and precision nutrition. Randomized controlled trials, observational studies, systematic reviews, meta-analyses, and mechanistic studies were included. Earlier landmark publications were retained when necessary to provide biological context or support fundamental concepts.

Eligible studies examined the relationships among dietary factors, gut microbiota, microbial metabolites, and biological aging. Particular emphasis was placed on studies involving older adults, advanced microbiome characterization, metabolomics, multi-omics approaches, and clinically relevant outcomes, including inflammation, metabolic health, cognitive function, muscle function, and frailty. Conference abstracts, editorials, non-English publications, and studies not directly addressing microbiota-mediated mechanisms relevant to healthy aging were excluded.

Evidence was synthesized thematically according to the major topics addressed in this review, including age-related changes in gut microbiota, dietary modulation of microbial function, microbiota-derived metabolites, gut–organ communication pathways, and their relevance to the biological hallmarks of aging. Where appropriate, greater weight was given to evidence from randomized controlled trials, systematic reviews, and meta-analyses than to findings derived from observational studies or experimental models. Because this is a narrative review, PRISMA methodology, formal risk-of-bias assessment, and other procedures specific to systematic reviews were not applied.

## 3. Age-Related Changes in Gut Microbiota Composition and Diversity

Aging is associated with alterations in gut microbial composition, diversity, and metabolic activity that influence immune, metabolic, and neurological homeostasis [14]. Because the gut microbiome is shaped by diet, physical activity, medication use, comorbidities, environmental exposures, and intrinsic aging processes, no single microbial signature reliably defines biological aging [15,16,17,18,19].

Cross-sectional and longitudinal studies show that the gut microbiome becomes increasingly heterogeneous with age, particularly after 65 years [20,21,22,23,24,25]. Reduced microbial diversity and depletion of SCFA-producing bacteria are frequently reported, but these findings are inconsistent across populations, indicating that microbiome aging is driven by multiple host and environmental factors rather than chronological age alone [22,26]. Among SCFAs, butyrate is of particular interest because it maintains epithelial barrier integrity; inhibits histone deacetylases (HDACs) and nuclear factor kappa B (NF-κB) signaling; activates G protein-coupled receptor 41 (GPR41), G protein-coupled receptor 43 (GPR43), and G protein-coupled receptor 109A (GPR109A); and promotes regulatory T-cell differentiation. [27]. 

Several microbial taxa, including *Faecalibacterium prausnitzii*, *Roseburia* spp., members of the Ruminococcaceae family, and *Akkermansia muciniphila*, have been associated with healthy aging [28,29,30]. *F. prausnitzii* is consistently depleted in frail older adults, whereas *A. muciniphila* has been associated with preserved barrier function and metabolic health [31,32,33,34,35,36,37]. Similarly, enrichment in *Christensenellaceae* has been linked to longevity, lower visceral adiposity, and greater microbial ecosystem stability [38,39,40,41]. Whether these associations reflect causal mechanisms or healthier host characteristics remains unresolved.

Lifestyle appears to influence the aging microbiome more strongly than chronological age. Western dietary patterns, low fiber intake, physical inactivity, polypharmacy, and antibiotic exposure are consistently associated with dysbiosis, whereas Mediterranean-style diets and regular physical activity are generally associated with more favorable microbial profiles [42,43,44,45,46,47,48,49,50,51,52,53,54,55,56,57,58,59,60,61,62,63,64,65].

**Figure 2:**
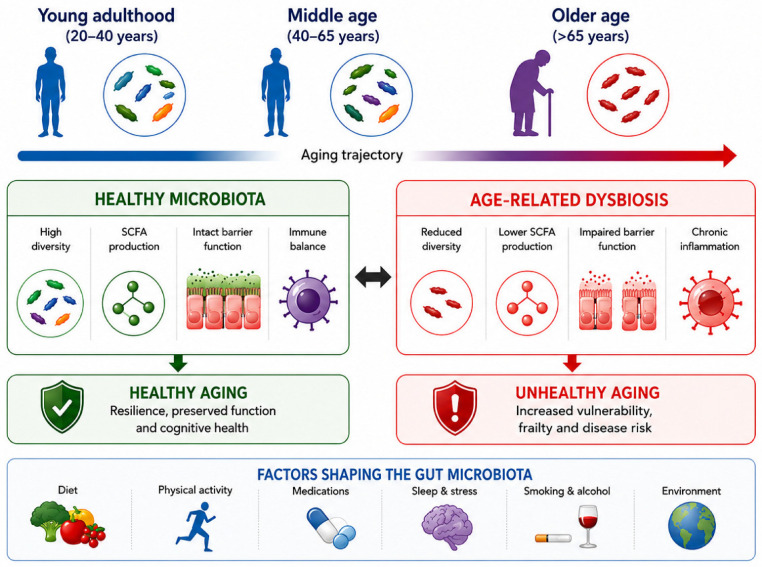
Age-related alterations in gut microbiota composition and function and their implications for healthy aging. The figure summarizes the dynamic changes in gut microbiota composition and function across the aging trajectory. Rather than representing a linear process, microbiome aging reflects the cumulative effects of lifestyle, environmental exposures, medication use, and host-related factors. Healthy aging is generally characterized by preservation of microbial diversity, SCFA production, intestinal barrier integrity, and immune homeostasis, whereas age-related dysbiosis is associated with reduced microbial diversity, impaired microbial metabolism, epithelial barrier dysfunction, and chronic low-grade inflammation. These alterations have been linked to gut–organ communication pathways that contribute to frailty, sarcopenia, cognitive decline, cardiometabolic disorders, and increased morbidity. Importantly, the figure represents a conceptual synthesis of current evidence and is intended to illustrate biological interactions rather than causal or quantitative relationships.

Frailty is one of the strongest clinical correlates of microbiome alterations. Frail older adults typically exhibit depletion of SCFA-producing bacteria together with enrichment in pro-inflammatory taxa [66,67,68,69]. Centenarian studies further emphasize the complexity of microbiome aging. Although exceptionally long-lived individuals frequently display distinct microbial and metabolic profiles, these are strongly influenced by genetics, diet, medication use, and geography [38,70,71,72,73,74]. Current evidence therefore suggests that preservation of microbial functional capacity, rather than the abundance of individual taxa, is more closely associated with healthy aging. Figure 2 summarizes the principal age-related alterations in gut microbiota composition and function, while representative microbial taxa, their proposed biological roles, and the strength of supporting evidence are presented in Table 1.

## 4. Polyphenols, Dietary Fiber, and Functional Nutrition as Modulators of Gut Microbiota

Diet is one of the major modifiable determinants of gut microbial composition and function. Landmark studies have demonstrated that age-related alterations in the gut microbiota are closely linked to dietary habits, frailty, and healthy aging. The intestinal microbiome is increasingly recognized as a functional metabolic organ that integrates immune, metabolic, and neuroendocrine signaling and thereby contributes to the maintenance of host homeostasis [77,78,79]. The multicenter NU-AGE trial demonstrated that long-term adherence to a Mediterranean diet increased the abundance of short-chain fatty acid (SCFA)-producing bacteria, reduced frailty, and improved overall health status in older adults [80]. Consistent with these findings, exceptionally long-lived individuals exhibit a distinct microbial ecosystem characterized by greater functional resilience and enrichment in health-associated taxa [81]. Collectively, current evidence indicates that reduced microbial diversity, impaired microbial metabolism, and chronic low-grade inflammation are recurrent features of biological aging. However, substantial methodological heterogeneity across studies highlights the need for standardized longitudinal and intervention studies to better define causal relationships [82]. Representative clinical and selected experimental studies investigating microbiota-targeted dietary strategies are summarized in Table 2.

The concept of functional nutrition recognizes that dietary bioactive compounds influence host physiology through interactions with the gut microbiota [98,99]. Dietary polyphenols and fermentable fiber modulate microbial metabolism and the production of bioactive metabolites that mediate host–microbiome communication [100,101,102,103]. Polyphenol–microbiota interactions are bidirectional: gut microorganisms convert poorly absorbed polyphenols into metabolites with greater bioavailability and biological activity, while polyphenols selectively shape microbial composition and metabolic function [104,105,106,107].

### 4.1. Polyphenols and Microbial Biotransformation

Polyphenols comprise a diverse group of plant-derived bioactive compounds, including flavonoids, phenolic acids, stilbenes, lignans, and tannins, abundant in fruits, vegetables, tea, coffee, cocoa, whole grains, nuts, and red wine [108,109]. Because only a small proportion is absorbed in the small intestine, most polyphenols reach the colon, where microbial biotransformation largely determines their biological activity [110,111].

Gut microorganisms convert polyphenols through deglycosylation, dehydroxylation, demethylation, reduction, decarboxylation, and ring cleavage, generating metabolites such as urolithins, phenolic acids, phenyl-γ-valerolactones, and dihydroresveratrol [74,105,106,112,113]. Compared with their parent compounds, these metabolites generally exhibit greater bioavailability and regulate pathways involved in mitochondrial function, oxidative stress, inflammation, and nutrient sensing. Experimental studies demonstrate activation of AMPK, SIRT1, and Nrf2; modulation of mTOR; and inhibition of NF-κB signaling and HDACs, thereby promoting mitophagy and antioxidant defense [114].

Microbial metabolism also contributes to substantial interindividual variability in responses to polyphenol-rich diets. The generation of metabolites depends on microbial composition and enzymatic capacity, giving rise to distinct metabotypes. A well-characterized example is equol production from soy isoflavones, which occurs only in a subset of individuals and has been associated with differential cardiometabolic responses [115,116,117,118,119].

Polyphenols also modify the intestinal ecosystem by enriching beneficial taxa, including *Bifidobacterium*, *Akkermansia*, and other SCFA-producing bacteria [101,120,121,122]. However, these effects vary according to baseline microbiome composition, dietary background, host genetics, and study design [123,124]. Consequently, microbial function and metabolite production may be more informative than taxonomic changes alone [120,121,122,123,124].

Ellagitannins derived from pomegranates, berries, and walnuts are hydrolyzed to ellagic acid and subsequently metabolized by gut bacteria, particularly *Gordonibacter* spp., into sequential urolithins (urolithins C, A, and B). Urolithin A induces mitophagy and improves mitochondrial quality control, thereby enhancing mitochondrial function and reducing oxidative stress. Dietary catechins are metabolized by intestinal microorganisms into phenyl-γ-valerolactones and subsequently into phenylpropionic acids. These metabolites interact with SCFA-mediated signaling and activate AMPK and Nrf2, enhancing antioxidant defense and metabolic homeostasis. Similarly, the soy isoflavone daidzin is hydrolyzed to daidzein and further converted by equol-producing gut bacteria into equol, a metabolite with high affinity for estrogen receptor β (ERβ) that exerts anti-inflammatory and vasculoprotective effects [125,126].

**Figure 3:**
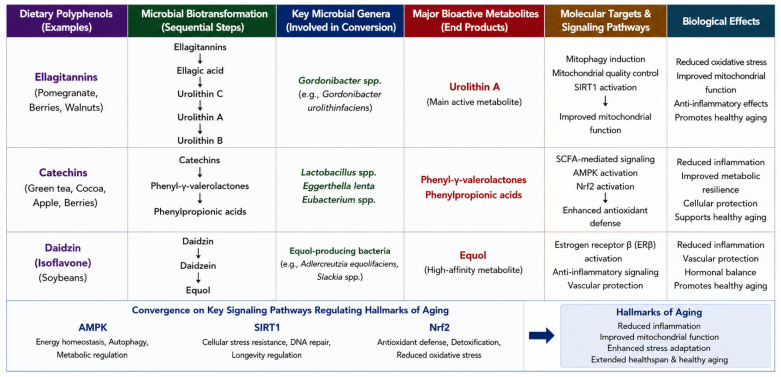
Dietary polyphenols are converted by gut microbiota into bioactive metabolites, including urolithin A, phenyl-γ-valerolactones, phenylpropionic acids, and equol. These metabolites regulate key signaling pathways (AMPK, SIRT1, and Nrf2), thereby influencing mitochondrial function, oxidative stress, inflammation, and multiple hallmarks of aging. Abbreviations: AMPK, AMP-activated protein kinase; ERβ, estrogen receptor beta; Nrf2, nuclear factor erythroid 2-related factor 2; SCFA, short-chain fatty acid; SIRT1, sirtuin 1.

The biological effects of dietary polyphenols are mediated primarily by microbiota-derived metabolites rather than by the parent compounds. These metabolites regulate key signaling pathways, including AMPK, SIRT1, and Nrf2, linking microbial metabolism to mitochondrial function, oxidative stress, inflammatory signaling, and metabolic homeostasis [125,126]. Consequently, gut microbial biotransformation represents a central mechanism through which dietary polyphenols influence multiple hallmarks of aging. The principal microbiota-mediated biotransformation pathways and their major molecular targets are summarized in Figure 3.

### 4.2. Dietary Fiber, Prebiotics, and SCFA Production

Dietary fiber comprises non-digestible carbohydrates that reach the colon, where they undergo microbial fermentation. Soluble fibers, including inulin, β-glucans, pectins, and fructooligosaccharides, exert prebiotic effects by selectively stimulating beneficial microorganisms, whereas insoluble fibers primarily increase stool bulk and accelerate intestinal transit [127,128,129,130].

Colonic fermentation of dietary fiber generates the SCFAs acetate, propionate, and butyrate, which function as key signaling metabolites linking microbial activity with host physiology [131]. SCFAs activate free fatty acid receptor 2 (FFAR2/GPR43), free fatty acid receptor 3 (FFAR3/GPR41), and G protein-coupled receptor 109A (GPR109A) while inhibiting histone deacetylases [132,133,134,135]. Among SCFAs, butyrate is of particular importance because it serves as the primary energy source for colonocytes, enhances tight-junction protein expression and mucin production, and supports epithelial oxygen homeostasis, thereby limiting intestinal permeability and the expansion of facultative anaerobic pathobionts [133,134,135].

Randomized dietary intervention studies consistently demonstrate enrichment in SCFA-producing taxa, particularly *Bifidobacterium*, *Faecalibacterium*, and *Roseburia*, following supplementation with resistant starch, inulin, or fructooligosaccharides [136,137,138,139]. Nevertheless, the magnitude of these responses varies according to baseline microbiota composition, habitual dietary patterns, intervention duration, and host-specific characteristics, highlighting the importance of personalized nutritional approaches.

The biological effects of SCFAs extend beyond intestinal homeostasis. Propionate stimulates glucagon-like peptide-1 (GLP-1) and peptide YY (PYY) secretion, whereas higher dietary fiber intake has been associated with improved insulin sensitivity, glycemic control, lipid metabolism, and reduced systemic inflammation [140]. In addition, interactions between microbial fermentation and bile acid metabolism may contribute to cardiometabolic regulation through activation of farnesoid X receptor (FXR) and Takeda G protein-coupled receptor 5 (TGR5), although direct causal evidence in humans remains limited [141].

### 4.3. Functional and Fermented Foods in Healthy Aging 

Functional and fermented foods represent an important component of microbiota-targeted nutritional strategies because they provide fermentation-derived bioactive metabolites and, in selected products, viable microorganisms capable of influencing host–microbiome interactions. Fermentation enhances the bioavailability of dietary phytochemicals while generating organic acids, bioactive peptides, exopolysaccharides, vitamins, and other microbial metabolites that contribute to intestinal ecosystem stability and metabolic homeostasis [16,142,143,144].

Traditional fermented foods, including yogurt, kefir, kimchi, sauerkraut, tempeh, miso, and kombucha, have been associated with increased gut microbial diversity, enrichment in SCFA-producing bacteria, improved intestinal barrier function, and modulation of immune homeostasis [142,145,146]. Proposed mechanisms include enhanced microbial production of SCFAs, alterations in bile acid metabolism, and regulation of AMPK, Nrf2, and NF-κB signaling, thereby contributing to attenuation of inflammaging and maintenance of metabolic homeostasis.

Among fermented foods, fermented dairy products provide the most robust clinical evidence. Randomized controlled trials have demonstrated that kefir and probiotic yogurt favorably modify gut microbiota composition, improve selected metabolic and inflammatory biomarkers, and support immune function. In older adults, high-protein fermented dairy products combined with resistance exercise increase muscle mass and physical performance, suggesting a potential role in the prevention of sarcopenia [146,147,148,149,150,151,152,153]. By contrast, evidence supporting independent effects on bone metabolism and fracture prevention remains limited [154].

Clinical evidence for fermented vegetables, soy-based fermented products, and kombucha is less consistent. Although several studies have reported beneficial gastrointestinal, cardiometabolic, or cognitive effects, most are limited by small sample sizes, heterogeneous interventions, short follow-up periods, and reliance on surrogate biomarkers rather than clinically relevant aging outcomes [155,156,157,158,159,160,161,162,163]. Consequently, adequately powered randomized controlled trials with standardized interventions and long-term clinical endpoints are required to establish the contribution of fermented foods to healthy aging. The major fermented foods, their bioactive constituents, microbiota-mediated mechanisms, and potential contributions to healthy aging are summarized in Table 3.

## 5. Microbiota-Derived Metabolites and Mechanisms of Healthy Aging

Gut microbial metabolism generates bioactive metabolites that mediate host–microbiome communication and regulate key biological processes involved in aging [165,166,167]. Age-related dysbiosis is accompanied by profound alterations in microbial metabolic activity, resulting in reduced production of health-promoting metabolites and increased generation of compounds associated with chronic inflammation and metabolic dysfunction [11,168,169]. Rather than acting solely as metabolic intermediates, microbiota-derived metabolites function as signaling molecules that integrate epigenetic, immunometabolic, and mitochondrial regulatory networks. Through modulation of nutrient-sensing and stress-response pathways, histone deacetylase (HDAC) activity, chromatin remodeling, and the mitochondrial quality control network—including mitophagy, mitochondrial fission, fusion, and biogenesis—they contribute to the maintenance of cellular homeostasis and biological resilience during aging. These integrated regulatory mechanisms influence mitochondrial function, autophagy, epithelial barrier integrity, immune homeostasis, and systemic metabolic adaptation [170,171,172,173]. The following sections summarize the major classes of microbiota-derived metabolites and critically evaluate their contribution to healthy aging.

### 5.1. Short-Chain Fatty Acids and Immunometabolic Regulation

SCFAs are central mediators linking microbial metabolism to host immunometabolic regulation. Beyond their established roles in intestinal homeostasis, SCFAs function as signaling molecules that coordinate nutrient sensing, immune responses, and cellular stress adaptation. Through receptor-dependent and epigenetic mechanisms, they modulate mitochondrial metabolism, inflammatory signaling, and cellular resilience, thereby influencing several hallmarks of biological aging [174,175,176,177,178,179,180]. In addition to receptor-mediated signaling, SCFAs inhibit histone deacetylases, thereby promoting epigenetic remodeling that regulates transcriptional programs involved in inflammatory resolution, mitochondrial biogenesis, and cellular stress resistance. These effects converge with activation of AMP-activated protein kinase and modulation of mechanistic target of rapamycin signaling, promoting autophagy and maintenance of mitochondrial quality control. Current evidence therefore supports SCFAs as biologically plausible mediators of healthy aging while highlighting the need for adequately powered longitudinal and randomized controlled studies to establish causal effects [81,181].

### 5.2. Secondary Bile Acids and Longevity Pathways

Beyond their established role in lipid digestion and fat absorption, bile acids function as important signaling molecules that regulate metabolic, immune, and mitochondrial homeostasis. Primary bile acids synthesized in the liver undergo microbial biotransformation in the intestine, generating secondary bile acids that serve as key mediators of bidirectional communication between the gut microbiota and the host [182].

The principal secondary bile acids—including deoxycholic acid (DCA), lithocholic acid (LCA), ursodeoxycholic acid (UDCA), and tauroursodeoxycholic acid (TUDCA)—exert their biological effects primarily through activation of the farnesoid X receptor (FXR) and the G protein-coupled bile acid receptor 1 (TGR5). Activation of these receptors regulates glucose and lipid metabolism, energy homeostasis, intestinal barrier integrity, and both innate and adaptive immune responses [168,183,184,185].

Age-associated gut microbial dysbiosis alters the composition of the bile acid pool, shifting it toward metabolites associated with chronic inflammation and metabolic dysfunction [186,187]. In contrast, ursodeoxycholic acid (UDCA) and tauroursodeoxycholic acid (TUDCA) have demonstrated cytoprotective, anti-apoptotic, and neuroprotective effects in experimental models. These bile acids attenuate endoplasmic reticulum stress, reduce oxidative damage, and preserve mitochondrial function, thereby contributing to the maintenance of cellular integrity and potentially slowing aging-related pathological processes [188,189].

Experimental studies have further demonstrated that the FXR–TGR5 signaling axis regulates nutrient-sensing, mitochondrial, and inflammatory pathways that are central to the biology of aging [189,190,191]. Emerging evidence also suggests that activation of FXR–TGR5 signaling promotes mitochondrial bioenergetics and immunometabolic adaptation through AMP-activated protein kinase (AMPK)- and peroxisome proliferator-activated receptor gamma coactivator-1α (PGC-1α)-dependent mechanisms. These signaling networks may enhance mitochondrial efficiency while suppressing pro-inflammatory macrophage activation and oxidative stress, thereby promoting metabolic resilience and supporting healthy aging [191,192,193].

### 5.3. Tryptophan Metabolism and Gut–Brain Communication

Tryptophan metabolism is a key pathway linking the gut microbiota with neuroimmune regulation and gut–brain communication [194]. As an essential amino acid, tryptophan is converted into serotonin, kynurenine metabolites, and microbiota-derived indole compounds, each contributing to distinct aspects of intestinal and neurological homeostasis [195].

Gut microorganisms generate bioactive metabolites, including indole, indole-3-aldehyde, indole-3-acetic acid, and tryptamine, which activate the aryl hydrocarbon receptor (AhR) and regulate mucosal immunity, epithelial barrier integrity, and host–microbiome homeostasis [196,197,198,199]. These metabolites also participate in gut–brain communication by influencing serotonin biosynthesis, vagal signaling, microglial function, and blood–brain barrier integrity [198,200].

Age-related dysbiosis shifts tryptophan metabolism toward increased indoleamine 2,3-dioxygenase (IDO1) activity and enhanced kynurenine pathway metabolism, resulting in greater production of pro-inflammatory and potentially neurotoxic metabolites [6,201]. 

Dietary polyphenols and fermentable fiber may partially restore microbial tryptophan metabolism and promote the generation of neuroprotective indole metabolites [8,202]. However, human intervention studies integrating microbiome profiling, metabolomics, and clinically relevant cognitive outcomes remain scarce. The principal microbiota-derived metabolites implicated in aging-related processes are summarized in Table 4.

## 6. Gut Microbiota and the Hallmarks of Aging

The geroscience framework proposes that aging results from interconnected biological mechanisms rather than independent pathological processes. Increasing evidence indicates that the gut microbiota functions as an upstream regulator of several hallmarks of aging through the production of bioactive metabolites that integrate nutrient sensing, immunometabolic regulation, mitochondrial homeostasis, and interorgan communication [12,28,203,204]. This concept has shifted microbiome research beyond descriptive taxonomic profiling toward functional characterization of microbial metabolism and its contribution to biological aging. Table 5 summarizes the current evidence linking microbiota-targeted nutritional strategies with individual hallmarks of aging and highlights areas where mechanistic and clinical evidence remain limited.

Among the recognized hallmarks of aging, the strongest microbiota-related evidence supports roles in inflammaging, dysregulated nutrient sensing, and mitochondrial dysfunction. Collectively, microbiota-derived metabolites integrate immune, metabolic, and mitochondrial signaling networks, providing a mechanistic link between diet and biological aging. Rather than modulating individual hallmarks in isolation, these metabolites appear to orchestrate extensive molecular crosstalk among nutrient-sensing pathways, mitochondrial quality control, epigenetic regulation, and immune signaling. This systems-level integration is consistent with the geroscience concept that the hallmarks of aging are mechanistically interconnected and may therefore be influenced simultaneously through microbiota-mediated metabolic signaling. In contrast, evidence for direct modulation of stem cell exhaustion, genomic instability, and altered intercellular communication remains largely experimental.

## 7. Gut Microbiota, Inflammaging, and Immunosenescence

Persistent activation of the innate immune system, driven by cellular senescence, mitochondrial dysfunction, and oxidative stress, is a central mechanism underlying inflammaging [204,205,206,207,208,209]. Age-related dysbiosis disrupts microbial metabolic function, amplifies inflammatory signaling, and contributes to the maintenance of chronic low-grade inflammation [210,211,212,213,214,215,216,217]. In parallel, microbiota-derived metabolites modulate adaptive immunity by regulating regulatory T-cell differentiation, T-helper cell polarization, and antigen-presenting cell function, although much of the supporting evidence is derived from experimental models [217,218,219].

Immunosenescence is characterized by depletion of naïve T- and B-cell populations, contraction of the T-cell receptor repertoire, and accumulation of functionally exhausted immune cells, resulting in impaired immune competence [220,221]. Because inflammaging and immunosenescence reinforce one another, preservation of microbial metabolic function has emerged as a biologically plausible strategy to maintain immune resilience during aging [222,223,224].

Microbiota-targeted nutritional interventions have demonstrated favorable effects on inflammatory and immune biomarkers in several clinical studies [5,224,225,226]. However, evidence supporting clinically meaningful improvements in infection risk, vaccine responsiveness, frailty, or healthspan remains limited and heterogeneous.

## 8. The Gut–Brain Axis in Cognitive Aging

**Figure 4:**
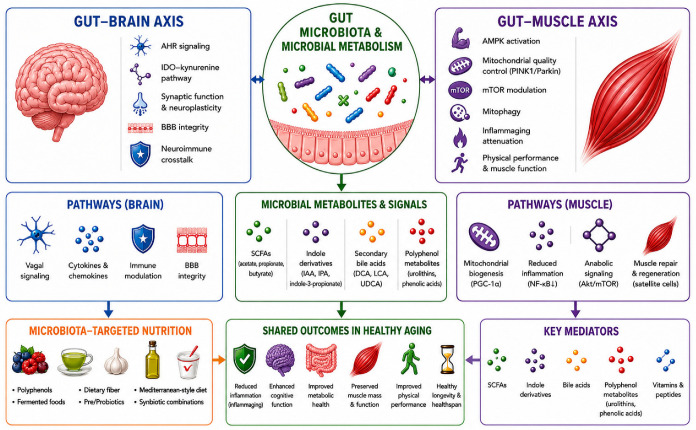
Conceptual overview of the interactions among the gut microbiota, microbiota-derived metabolites, and the gut–brain and gut–muscle axes in healthy aging. Microbiota-derived metabolites influence neuroimmune signaling, blood–brain barrier integrity, mitochondrial function, muscle metabolism, and systemic inflammation, thereby contributing to cognitive and physical resilience during aging. The figure summarizes current mechanistic evidence and is intended as a conceptual framework rather than a representation of causal or quantitative relationships. Abbreviations: BBB, blood–brain barrier; GBA, gut–brain axis; GMA, gut–muscle axis; SCFAs, short-chain fatty acids.

The gut–brain axis is a bidirectional communication network linking the gastrointestinal tract, gut microbiota, and central nervous system through neural, endocrine, immune, and metabolic pathways [227]. Microbiota-derived metabolites regulate neuroimmune signaling, blood–brain barrier integrity, neurotransmitter metabolism, and microglial function, thereby providing mechanistic links between the gut microbiota and cognitive aging [228,229,230]. Figure 4 summarizes the principal molecular and physiological interactions within the gut–brain and gut–muscle axes, highlighting the proposed roles of microbiota-derived metabolites in healthy aging.

The gut microbiota influences brain function through microbiota-derived metabolites, immune and endocrine signaling pathways, and vagal communication [6,231]. Age-related dysbiosis is characterized by impaired microbial metabolic function, chronic low-grade inflammation, and increased levels of lipopolysaccharide (LPS), trimethylamine N-oxide (TMAO), and kynurenine metabolites, all of which have been associated with neuroinflammation and cognitive decline [5,195,198,216,232,233,234,235,236,237,238,239,240,241,242,243,244]. Microbiota-targeted nutritional interventions may improve microbial metabolic function and selected cognitive and inflammatory biomarkers [7,235,236]. However, evidence supporting sustained clinical benefits for cognitive aging remains limited, as most intervention studies are small, heterogeneous, and rely primarily on surrogate biomarkers rather than clinically relevant outcomes [237,245,246,247].

### 8.1. Microbial Metabolites and Neuroinflammation

Microbiota-derived metabolites are key mediators of the gut–brain axis, contributing to neuroimmune regulation, blood–brain barrier integrity, and neuronal homeostasis [241,248,249,250]. Age-related dysbiosis alters microbial metabolic function, promoting neuroinflammation and potentially contributing to cognitive decline [243,247,248,249,250,251,252,253,254,255]. Current evidence suggests that microbial metabolic function is more closely associated with cognitive aging than taxonomic composition alone, emphasizing the importance of functional rather than compositional microbiome profiling. Future research integrating microbiome profiling, metabolomics, and neuroimaging will be essential to identify microbial metabolic signatures associated with healthy brain aging.

### 8.2. Polyphenols and Cognitive Resilience

Polyphenol-rich dietary patterns have consistently been associated with better cognitive performance and a lower risk of cognitive decline in observational studies [256,257]. The neuroprotective effects of polyphenols largely depend on microbial biotransformation (Section 4.1), which converts parent compounds into metabolites with greater bioavailability and biological activity [114]. These metabolites regulate interconnected molecular pathways involved in mitochondrial quality control, oxidative stress, neuroinflammation, synaptic plasticity, and brain-derived neurotrophic factor (BDNF) signaling [258,259,260,261,262,263,264].

Accumulating evidence indicates that the biological effects of polyphenols are determined not only by dietary intake but also by the metabolic capacity of the gut microbiota. Interindividual differences in microbial enzymatic activity give rise to distinct metabotypes, which may partly explain the marked heterogeneity observed in clinical responses to polyphenol interventions [265,266].

## 9. The Gut–Muscle Axis and Sarcopenia

Observational studies consistently demonstrate associations between gut microbiota dysbiosis and reduced muscle strength, impaired physical performance, frailty, and sarcopenia [267,268,269,270]. Accumulating mechanistic evidence indicates that microbial metabolic function, rather than taxonomic composition alone, is a key determinant of the gut–muscle axis through its regulation of mitochondrial bioenergetics, insulin sensitivity, skeletal muscle protein metabolism, and inflammatory and anabolic signaling pathways [271,272,273,274,275,276,277,278,279].

The gut–muscle axis is jointly modulated by physical activity and diet. Regular exercise enhances microbial diversity and metabolic capacity, while exercise-induced myokines influence the functional activity of the gut microbiota. Concurrently, dietary patterns rich in high-quality protein, fermentable fiber, and plant-derived bioactive compounds support both skeletal muscle metabolism and microbial homeostasis, reinforcing the bidirectional communication between skeletal muscle and the gut microbiota [280,281,282,283,284]. Although microbiota-targeted nutritional interventions and microbiome-modulating strategies have shown promising results in experimental models, clinical evidence in humans remains limited [285].

## 10. Future Directions in Microbiota-Targeted Geroscience

The gut microbiota has emerged as a central target in geroscience because it integrates dietary, metabolic, immune, and microbial signaling pathways that collectively regulate biological aging [286]. Future research should move beyond descriptive taxonomic profiling toward functional characterization of host–microbiome interactions and identification of molecular mechanisms directly linked to healthspan.

The integration of metagenomics, metatranscriptomics, metabolomics, proteomics, lipidomics, and single-cell technologies provides unprecedented opportunities to characterize microbial function and host–microbiome interactions with greater precision [286,287,288]. Combined with standardized clinical phenotyping, these approaches are expected to facilitate the identification of biomarkers and molecular pathways associated with healthy aging.

The advancement of precision nutrition will depend on integrating microbiome composition and functional capacity with host genetics, metabolic phenotype, immune status, dietary habits, and lifestyle factors [171,289,290,291,292]. In parallel, microbiome-derived biomarkers, metabolite profiling, and artificial intelligence- and machine learning-based analytical approaches offer new opportunities to improve the assessment of biological aging, frailty, cognitive decline, and cardiometabolic risk. Publicly available resources, including the Human Microbiome Project (HMP), MGnify, the Unified Human Gastrointestinal Genome (UHGG) catalog, GMrepo, the Global Natural Products Social Molecular Networking (GNPS) platform, and the Human Metabolome Database (HMDB), provide valuable datasets for biomarker discovery, multi-omics integration, and validation of microbiota-targeted nutritional interventions, thereby facilitating reproducible analyses and accelerating the development of precision nutrition strategies for healthy aging.

**Figure 5:**
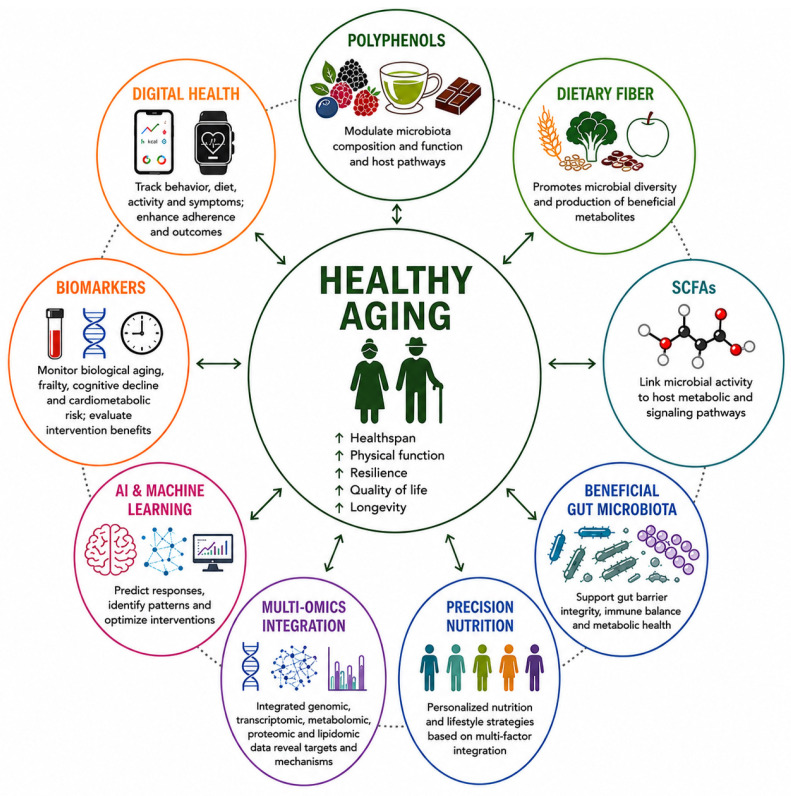
Conceptual framework illustrating emerging microbiota-based strategies to promote healthy aging through dietary interventions (polyphenols and dietary fiber), microbiota-derived metabolites, precision nutrition, multi-omics, artificial intelligence, digital health, biomarker discovery, and microbiome-targeted therapeutics. These approaches aim to improve healthspan, resilience, cognitive function, and longevity. The figure is conceptual and does not imply causal or quantitative relationships. Abbreviations: AI, artificial intelligence; HMP, Human Microbiome Project; HMDB, Human Metabolome Database; GNPS, Global Natural Products Social Molecular Networking; MGnify, Microbiome Analysis Resource; SCFAs, short-chain fatty acids; UHGG, Unified Human Gastrointestinal Genome.

Emerging microbiome-based interventions—including probiotics, prebiotics, synbiotics, postbiotics, engineered microbial therapeutics, and fecal microbiota transplantation—may complement dietary strategies by targeting microbial function in addition to microbial composition [237,293,294,295,296]. Future progress in microbiota-targeted geroscience will therefore depend on well-characterized longitudinal cohorts, mechanistic human intervention studies, standardized analytical methodologies, validated biomarkers, and integrated multi-omics approaches linking microbial function with clinically meaningful aging outcomes (Figure 5).

## 11. Limitations of Current Evidence

Despite growing interest in the role of the gut microbiota in healthy aging, important methodological limitations continue to restrict interpretation of the available evidence and its clinical translation.

Substantial heterogeneity across studies remains a major challenge. Differences in study populations, dietary exposures, medication use, antibiotic exposure, intervention duration, sequencing technologies, sample processing, bioinformatic pipelines, and clinical endpoints limit reproducibility and comparability. In addition, polyphenol and fermentable fiber interventions vary considerably in composition, dosage, bioavailability, and adherence.

Most evidence is derived from observational studies and cannot establish causality. Randomized controlled trials remain limited by small sample sizes, short follow-up, and reliance on surrogate biomarkers rather than clinically relevant aging outcomes. Consequently, evidence for microbiota-targeted interventions to prevent frailty, sarcopenia, cognitive decline, disability, and mortality remains limited. Publication bias may also contribute to an overrepresentation of positive findings.

Interpretation is complicated by substantial interindividual variability. Gut microbial composition is influenced by genetics, age, diet, medication use, physical activity, environmental exposures, socioeconomic factors, and geographic location, limiting the generalizability of microbial signatures across populations.

Assessment of dietary exposure is limited because most studies rely on self-reported dietary questionnaires. In addition, the polyphenol content and bioavailability of foods vary according to food source, processing, food matrix, and host metabolism.

Finally, although experimental studies support plausible biological mechanisms, it remains unclear whether many microbiome alterations represent causal drivers, consequences of aging, compensatory responses, or biomarkers of disease. Methodological heterogeneity, limited standardization, and insufficient clinical evidence continue to limit clinical translation.

## 12. Conclusions

The gut microbiota has emerged as a central mediator linking nutrition with biological aging through its effects on microbial metabolism, immune regulation, and host signaling pathways. Dietary polyphenols and fermentable fiber modulate microbial function and the production of bioactive metabolites, thereby influencing multiple hallmarks of aging, including inflammaging, mitochondrial homeostasis, immunometabolic regulation, and gut–organ communication. Collectively, current evidence supports a systems-level model in which microbiota-derived metabolites integrate immune, metabolic, epigenetic, and mitochondrial signaling networks, coordinating biological resilience during aging. Importantly, emerging evidence suggests that microbial functional capacity and metabolite production, rather than taxonomic composition alone, constitute the principal mechanistic link between nutrition and biological aging. This functional perspective shifts the focus of microbiome research from descriptive microbial profiling toward mechanistic characterization of host–microbiome interactions.

Future progress will depend on standardized microbiome methodologies, integrated multi-omics approaches, and well-designed longitudinal and intervention studies capable of establishing causal relationships between microbiota-targeted nutrition and healthy aging. These advances may enable evidence-based personalized nutritional strategies to improve healthspan.

## Figures and Tables

**Table 1:** Gut microbial taxa associated with healthy aging, longevity, and frailty-related dysbiosis: proposed biological functions and representative evidence.

Microbial Taxa	Association with Healthy Aging/Longevity	Association with Frailty/Dysbiosis	Proposed Biological Mechanisms	Strength of Evidence	Representative Evidence
*Faecalibacterium prausnitzii*	↑ Enriched in healthy older adults and longevity-associated microbiomes	↓ Consistently depleted in frailty	Major butyrate producer; maintenance of epithelial barrier integrity; experimental evidence supports HDAC inhibition, NF-κB suppression, and regulatory T-cell induction	High	Consistently depleted in frail older adults across multiple observational cohorts
*Roseburia* spp.	↑ Associated with metabolically healthy aging	↓ Reduced in frailty and inflammaging	Butyrate production; maintenance of mucosal homeostasis; anti-inflammatory effects	Moderate	Lower abundance reported in frailty and unhealthy aging phenotypes
*Coprococcus* spp./*C. eutactus*	↑ Associated with healthy aging and greater microbial diversity	↓ Negatively associated with frailty	SCFA production; potential gut–brain metabolic signaling	Low–Moderate	Reduced abundance associated with frailty in community-dwelling older adults
*Akkermansia muciniphila*	↑ Enriched in healthy aging and centenarians	Variable; depletion associated with metabolic dysfunction	Mucin degradation; maintenance of epithelial barrier integrity; metabolic regulation	Moderate	Enriched in several longevity cohorts, although findings remain heterogeneous
*Christensenellaceae*	↑ Associated with longevity and favorable metabolic phenotype	↓ Reduced in frailty-associated microbiomes	Maintenance of microbial ecosystem stability and metabolic homeostasis	Moderate	Recurrently enriched in centenarians and exceptionally long-lived individuals
*Prevotella* spp./*P. copri*	↑ Associated with fiber-rich diets and healthy community-dwelling older adults	↓ Depleted in frailty	Fermentation of complex carbohydrates; modulation of glucose metabolism	Low–Moderate	Loss of *Prevotella*-rich enterotypes associated with frailty in observational studies
*Bifidobacterium* spp.	↑ Associated with intestinal homeostasis and healthy immune aging	↓ Age-related decline in several cohorts	Production of beneficial metabolites; immune modulation; maintenance of intestinal integrity	Moderate	Declining abundance reported in multiple aging cohorts
*Alistipes shahii*	↑ Associated with non-frail phenotype	↓ Reduced in frailty	Potential anti-inflammatory and metabolic functions	Low	Lower abundance consistently observed in frailty meta-analyses
*Eggerthella lenta*	↓ Generally low abundance in healthy aging	↑ Enriched in frailty and chronic inflammatory states	Pro-inflammatory metabolism; potential modulation of Th17-related immune responses	Low	Increased abundance associated with frailty and inflammaging
*Enterobacteriaceae*/*Klebsiella* spp.	↓ Low abundance in healthy aging	↑ Expansion associated with dysbiosis and endotoxemia	LPS production; epithelial barrier disruption; inflammatory activation	Moderate	Increased abundance repeatedly observed in aging-associated dysbiosis
*Bacteroides fragilis*	Context-dependent	Certain strains associated with frailty-related dysbiosis	Context-dependent immunomodulatory activity; selected strains linked to inflammatory and TMAO-related pathways	Low	Associations reported in frailty cohorts but remain inconsistent
*Clostridium hathewayi*	↓ Not characteristic of healthy aging	↑ Associated with frailty and metabolic disorders	Pro-inflammatory metabolic activity; possible involvement in TMA-related pathways	Low	Reported in observational studies of frailty and cardiometabolic dysfunction

Sources: Based on data summarized from references [16,31,40,68,69,72,73,74,75,76]. Abbreviations: HDAC, histone deacetylase; LPS, lipopolysaccharide; NF-κB, nuclear factor kappa B; SCFA, short-chain fatty acid; TMAO, trimethylamine N-oxide. Strength of evidence: High = consistent findings across multiple observational cohorts supported by mechanistic evidence; Moderate = reproducible associations across several studies but limited interventional confirmation; Low–Moderate = supportive but heterogeneous evidence; Low = preliminary or inconsistent evidence requiring further validation. Symbols: ↑ indicates enrichment or a positive association, whereas ↓ indicates depletion or a negative association.

**Table 2:** Representative randomized controlled trials, dietary intervention studies, systematic reviews, meta-analyses, and selected experimental studies investigating the effects of microbiota-targeted dietary strategies on gut microbiota, microbial metabolites, and clinically relevant outcomes (2016–2026).

Study	Study Type	Population	Intervention	Duration	Main Gut Microbiota Findings	Main Clinical/Metabolic Outcomes
Del Bo’ et al., 2021 [82]	Randomized controlled trial	Older adults (≥60 y) with increased intestinal permeability	Polyphenol-rich diet	8 weeks	↑ *Faecalibacterium* and butyrate-producing taxa	↓ zonulin, ↓ blood pressure, improved gut barrier function
Sergeev et al., 2020 [83]	Randomized controlled trial	Overweight/obese adults	Synbiotic + low-carbohydrate/high-protein diet	3 months	↑ *Bifidobacterium* and *Lactobacillus*	↓ HbA1C; improved gut microbiota composition
Kim et al., 2020 [84]	Randomized crossover trial	Healthy older adults	*Helianthus tuberosus* (Jerusalem artichoke) fiber	1 week	↓ *Ruminococcus* abundance	Improved postprandial glucose response
Kiewiet et al., 2021 [85]	Dietary intervention study	Adults aged 55–80 years	Chicory long-chain inulin	2 months	↑ microbial diversity; ↑ *Bifidobacterium* and *Anaerostipes hadrus*	Limited immune changes; ↓ isobutyric acid
Alfa et al., 2018 [86]	Randomized controlled trial	Older and middle-aged adults	Resistant starch prebiotic (MSPrebiotic^®^)	12 weeks	↑ *Bifidobacterium*; ↑ butyrate production	Improved SCFA profile; reduced stool softener use
Chung et al., 2016 [87]	Human dietary intervention study	Healthy adults	Inulin or apple pectin supplementation	12 days	Inulin: ↑ *Faecalibacterium prausnitzii* and *Anaerostipes hadrus*; pectin: ↑ *Eubacterium eligens*	↑ SCFA production
Machado et al., 2019 [88]	Randomized double-blind placebo-controlled trial	Overweight adults	Yacon flour	6 weeks	Prebiotic/FOS-related microbiota modulation	↓ body weight; ↓ waist circumference
Pagliai et al., 2020 [89]	Randomized crossover clinical trial (CARDIVEG)	Adults with low-to-moderate cardiovascular risk	Mediterranean diet vs vegetarian diet	3 months	↑ SCFA-associated genera	Improved cardiometabolic parameters
Galié et al., 2021 [78]	Randomized crossover clinical trial	Adults with metabolic syndrome	Mediterranean diet vs. nut supplementation	2 months	Modulation of SCFA-associated taxa	Improved insulin resistance
Meslier et al., 2020 [90]	Controlled dietary intervention study	Overweight/obese adults	Isocaloric Mediterranean diet	8 weeks	↑ *Faecalibacterium prausnitzii*	↓ plasma cholesterol; improved inflammatory profile
Rinott et al., 2022 [79]	Randomized controlled trial	Adults with abdominal obesity/dyslipidemia	Green Mediterranean diet	6 months	↑ *Prevotella*; altered BCAA pathways	↓ body weight; ↓ HOMA-IR
García-Cordero et al., 2023 [91]	Randomized controlled trial	Healthy aging adults	Cocoa flavanols and red berry anthocyanins	12 weeks	↑ SCFA-related fermentation; ↓ TMAO	Improved endothelial function
Guevara-Cruz et al., 2020 [92]	Randomized controlled trial	Adults with obesity and insulin resistance	Genistein	2 months	↑ *Akkermansia muciniphila*	↑ AMPK activation; improved insulin sensitivity
Depommier et al., 2019 [32]	Randomized double-blind placebo-controlled trial	Overweight/obese insulin-resistant adults	*Akkermansia muciniphila* supplementation	3 months	↑ fecal *Akkermansia*	↓ cholesterol; improved insulin sensitivity
Toderescu et al., 2026 [93]	Narrative review	Adults with healthy or metabolic conditions	Dietary polyphenols	—	↑ Beneficial taxa	Improved metabolic profile
González-Gómez et al., 2025 [94]	Systematic review	Overweight/obese adults	Polyphenol-rich interventions	—	↑ butyrate; ↑ beneficial taxa	↓ LPS; improved inflammatory profile
Mao et al., 2025 [95]	Systematic review	Individuals with overweight or obesity	Dietary polyphenols	5 weeks	↓ *Firmicutes*; ↓ *Proteobacteria*	↓ LPS; improved inflammatory profile
Marino et al., 2024 [96]	Systematic review	Adults with healthy or metabolic conditions	Berry-derived polyphenols	—	↑ *Bifidobacterium*; ↑ *Akkermansia*	Improved cardiometabolic profile
Ghosh et al., 2020 [80]	Multicenter randomized controlled trial	Adults aged 65–79 years	Mediterranean diet	12 months	↑ SCFA-producing taxa	↓ frailty; improved cognition
Dell’Olio et al., 2024 [97]	In vitro fermentation study	Lean and obese fecal microbiota	Apple, apple pectin and cellulose fibers	24 h	↑ *Akkermansia*, *Bifidobacterium*	Improved microbial metabolic profile

Abbreviations: AMPK, AMP-activated protein kinase; BCAA, branched-chain amino acid; FOS, fructooligosaccharides; HbA1c, glycated hemoglobin; HOMA-IR, homeostatic model assessment of insulin resistance; LPS, lipopolysaccharide; SCFA, short-chain fatty acid; TMAO, trimethylamine N-oxide. ↑ indicates an increase; ↓ indicates a decrease.

**Table 3:** Fermented foods and potential mechanisms relevant to healthy aging.

Fermented Food Category	Major Bioactive Components	Proposed Microbiota-Related Mechanisms	Potential Health Outcomes	Strength of Evidence
Yogurt	*Lactobacillus*, *Bifidobacterium*, bioactive peptides	Modulation of gut microbiota composition; immune regulation	Improved immune function; support of muscle health	Moderate–High
Kefir	Lactic acid bacteria (LAB), yeasts, exopolysaccharides	Increased microbial diversity; modulation of inflammatory responses	Improved metabolic health; potential benefits for bone metabolism	Moderate
Kimchi	LAB, phytochemicals, dietary fiber	Modulation of gut microbiota; increased microbial metabolite production	Gastrointestinal and cardiometabolic benefits	Low–Moderate
Sauerkraut	LAB, organic acids	Support of intestinal homeostasis	Digestive and immune support	Low
Tempeh/fermented soy	Isoflavone-derived metabolites, bioactive peptides	Gut–brain axis modulation; microbial metabolism of isoflavones	Potential support of cognitive function	Low–Moderate
Kombucha	Organic acids, microbial metabolites	Proposed modulation of microbial and metabolic pathways	Potential improvement in glycemic control	Low

Sources: Based on data summarized from references [146,147,148,149,150,151,152,153,154,155,156,157,158,159,160,161,162,163,164]. Abbreviations: LAB, lactic acid bacteria. Strength of evidence: High = supported by multiple randomized controlled trials and/or meta-analyses; Moderate = supported by several human intervention studies but requiring further confirmation; Low–Moderate = based on limited clinical evidence with supportive mechanistic studies; Low = primarily supported by preliminary clinical and/or experimental evidence.

**Table 4:** Major gut microbiota-derived metabolites involved in healthy aging: principal molecular mechanisms, biological functions, and strength of current evidence.

Metabolite	Major Microbial Sources/Precursors	Principal Molecular Targets and Mechanisms	Major Biological Functions	Clinical Relevance for Healthy Aging	Strength of Evidence
Butyrate	*Faecalibacterium prausnitzii*, *Roseburia* spp., *Eubacterium rectale*; fermentation of dietary fiber and resistant starch	HDAC inhibition; FFAR2 (GPR43), FFAR3 (GPR41), and GPR109A activation; AMPK signaling	Maintenance of epithelial barrier integrity, immune regulation, mitochondrial metabolism	Associated with reduced inflammaging, improved insulin sensitivity, and preservation of intestinal barrier function	High
Propionate	*Bacteroides* spp., *Veillonella* spp.; dietary fiber fermentation	FFAR2/FFAR3 signaling; modulation of hepatic glucose and lipid metabolism	Regulation of energy homeostasis and immune responses	Associated with improved metabolic homeostasis and reduced systemic inflammation	Moderate
Acetate	Broad anaerobic gut microbiota; carbohydrate fermentation	Acetyl-CoA metabolism; peripheral substrate utilization; gut–brain metabolic signaling	Energy metabolism and host–microbiome communication	Supports metabolic flexibility and intestinal homeostasis	Moderate
Indole-3-propionic acid (IPA)	*Clostridium sporogenes* and other tryptophan-metabolizing bacteria	AhR activation; Nrf2-mediated antioxidant defense; epithelial barrier protection	Neuroprotection, antioxidant activity, immune regulation	Associated with improved cognitive resilience and reduced neuroinflammation	Moderate
Urolithin A	Microbial metabolism of ellagitannins and ellagic acid (berries, walnuts, pomegranate)	Activation of PINK1/PARKIN- and BNIP3-mediated mitophagy; mitochondrial quality control	Enhancement of mitochondrial function and muscle homeostasis	Experimental and early clinical evidence suggests improved mitochondrial function and physical performance	Moderate
Equol	Microbial conversion of soy isoflavones by equol-producing microbiota	Estrogen receptor modulation; antioxidant and anti-inflammatory signaling	Vascular protection, metabolic regulation, neuroprotection	Associated with improved cardiometabolic and cognitive outcomes in equol producers	Moderate
Secondary bile acids	Microbial transformation of primary bile acids; *Clostridium* clusters and related taxa	FXR and TGR5 signaling; regulation of glucose, lipid, and energy metabolism	Metabolic regulation, immune modulation, host–microbiome signaling	Altered bile acid profiles are associated with metabolic aging and longevity-related phenotypes	Moderate
Polyamines (spermidine, spermine)	Host and microbial synthesis; dietary and microbial sources	Autophagy induction; AMPK activation; mTOR inhibition	Maintenance of proteostasis, cellular stress resistance	Associated with longevity, reduced inflammation, and preserved cognitive function	Moderate
Trimethylamine N-oxide (TMAO)	Microbial metabolism of choline and carnitine to trimethylamine (TMA), followed by hepatic oxidation	Endothelial dysfunction; inflammatory signaling; foam-cell formation	Cardiometabolic and vascular regulation	Elevated circulating levels are associated with cardiovascular disease and adverse cardiometabolic aging; causal role remains under investigation	Moderate
Imidazole propionate	Histidine-metabolizing gut microbiota	Impairment of insulin signaling; activation of mTORC1-related pathways	Glucose dysregulation and metabolic dysfunction	Associated with insulin resistance, obesity, and age-related metabolic disease	Low–Moderate

Sources: Based on representative evidence summarized from references [11,40,177,178,179,182,183,184,185,186,187,188,189,190,191,192,193,194,195,196,197,198,199,200,201,202]. Abbreviations: AhR, aryl hydrocarbon receptor; AMPK, AMP-activated protein kinase; BNIP3, BCL2 interacting protein 3; FFAR, free fatty acid receptor; FXR, farnesoid X receptor; GPR, G protein-coupled receptor; HDAC, histone deacetylase; IPA, indole-3-propionic acid; mTOR, mechanistic target of rapamycin; Nrf2, nuclear factor erythroid 2-related factor 2; PARKIN, parkin RBR E3 ubiquitin protein ligase; PINK1, PTEN-induced kinase 1; TGR5, Takeda G protein-coupled receptor 5; TMA, trimethylamine; TMAO, trimethylamine N-oxide. Strength of evidence: High = supported by multiple observational studies together with mechanistic and human intervention evidence; Moderate = supported by consistent mechanistic and observational evidence with limited human intervention data; Low–Moderate = promising but heterogeneous evidence requiring further validation.

**Table 5:** Potential interactions between microbiota-targeted nutrition and selected hallmarks of aging.

Hallmark of Aging	Polyphenol-Mediated Modulation	Fiber-Mediated Modulation	Representative Microbiota-Derived Mediators
Inflammaging [165,174,177]	Strong	Strong	Butyrate, propionate
Cellular senescence [114,180,204,205]	Moderate	Emerging	Urolithin A, butyrate
Mitochondrial dysfunction [126,180]	Moderate–Strong	Moderate	Urolithin A, SCFAs
Stem cell exhaustion	Emerging	Emerging	SCFAs, indole derivatives
Dysregulated nutrient sensing [126,141,180]	Moderate	Moderate	SCFAs
Impaired intercellular communication [170,182,193]	Moderate	Moderate	SCFAs, secondary bile acids
Gut barrier dysfunction [82,132,176,181,199]	Strong	Strong	Butyrate, indole derivatives
Immunosenescence [11,178,195,196]	Strong	Moderate-Strong	SCFAs, tryptophan metabolites

Abbreviations: SCFAs, short-chain fatty acids. Evidence strength reflects the quality and consistency of mechanistic, observational, and human intervention studies. Strong = consistent mechanistic and clinical evidence; Moderate = reproducible evidence with limited human confirmation; Limited = preliminary or inconsistent evidence; Emerging = biologically plausible mechanisms requiring further validation in well-designed human studies.

## Data Availability

No new data were created or analyzed in this study. Data sharing is not applicable to this article.
